# Novel small molecule inhibitor of GPR68 attenuates endothelial dysfunction and lung injury caused by bacterial lipopolysaccharide

**DOI:** 10.1038/s41598-025-02582-y

**Published:** 2025-11-05

**Authors:** Pratap Karki, Yunbo Ke, Chen-Ou Zhang, Kamoltip Promnares, Yue Li, Charles H. Williams, Konstantin G. Birukov, Charles C. Hong, Anna A. Birukova

**Affiliations:** 1 UMSOM Lung Biology Program, Division of Pulmonary and Critical Care, Department of Medicine, University of Maryland School of Medicine, 20 Penn Street, HSF-2, Room S143, Baltimore, MD 21201 USA; 2 Department of Anesthesiology, University of Maryland School of Medicine, Baltimore, MD 21201 USA; 3 Division of Cardiovascular Medicine, Department of Medicine, University of Maryland School of Medicine, Baltimore, MD 21201 USA; 4 Present Address: Department of Medicine, Michigan State University College of Human Medicine, East Lansing, MI 48824 USA

**Keywords:** Endothelial permeability, Lung injury, Inflammation, LPS, CRX-527, GPR68, OGM-8345, Cell biology, Diseases

## Abstract

The treatment of acute respiratory distress syndrome (ARDS) associated with high mortality remains a major global clinical challenge. Role of pulmonary endothelial dysfunction in uncontrolled lung inflammation and alveolar flooding is increasingly recognized. GPR68 is a member of proton-sensing G protein-coupled receptor family and highly expressed in pulmonary vascular endothelial cells (EC). Although GPR68 has been implicated in flow-induced, EC-dependent vasodilation, its role in endothelial dysfunction caused by ARDS-related insults remains unknown. Herein, we assessed the role of a first-in-class GPR68 inhibitor ogremorphin (OGM-8345) discovered by our group in modulating pulmonary EC dysfunction and lung injury in mice caused by bacterial lipopolysaccharide LPS. GPCR activation Tango assay showed that LPS-induced EC barrier failure was associated with increased GPR68 activity and was suppressed by OGM-8345. OGM-8345, but not GPR4 inhibitor NE52-QQ57, strongly attenuated LPS-induced paracellular gap formation, adherence junction disassembly and EC hyper-permeability, suppressed activation of the NF-kB pathway, and blocked LPS-induced protein expression of EC adhesion molecules ICAM-1 and VCAM-1 and transcriptional activation of cytokines and chemokines. Beneficial effects of OGM-8345 were also observed in the clinically relevant post-treatment model of EC dysfunction caused by LPS or synthetic TLR4 activator, CRX-527. Protective effects of OGM-8345 were observed in lung EC from both macrovascular and microvascular beds. In vivo, OGM-8345 ameliorated vascular leak and inflammation in mouse lungs exposed to LPS, as determined by Evans blue extravasation assay, total cell and protein content in bronchoalveolar lavage samples, and mRNA levels of inflammatory marker genes in lung tissue. Taken together, these findings uncover a role of GPR68 in propagation of EC barrier compromise and inflammation caused by bacterial agents and suggest a therapeutic potential of GPR68-selective inhibitor in improving EC dysfunction and lung injury.

## Introduction

Disruption of endothelial cell (EC) barrier and activation of inflammatory response represent two major pathological hallmarks of acute respiratory distress syndrome (ARDS) that still remains a major clinical concern in severely ill patients associated with 30–40% mortality^1,2^. ARDS characterized by severe pulmonary EC dysfunction is also a major mortality factor of the current COVID-19 pandemic, urging the need of new therapeutics to restore endothelial function^3–5^. Although direct mechanisms of lung injury caused by infectious agents and host–pathogen immune responses remain the main focus of current investigations, the role of confounding factors that may affect severity of disease remain much less understood.

Inflammation is associated with acidification of local tissue microenvironment^6^, but precise role of acidosis in triggering inflammatory response is not known. A subfamily of G protein-coupled receptors (GPCRs) encompassing GPR4, GPR65 or T-cell death associated gene 8 (TDAG8), GPR68 or ovarian cancer GPCR1 (OGR1), and GPR132 function as proton sensors^7,8^. The expression of all four pH-sensing GPCRs have been reported in various tissues and cell types, and alteration of their expression during various pathological conditions, largely based on the data obtained from mRNA expression analysis [Reviewed in^9,10^].These proton-sensing GPCRs have long been known to regulate various aspects of tumor biology, cardiovascular physiology, and respiratory diseases such as asthma^10^. More recently, GPR4 has been suggested to modulate endothelial permeability and inflammation. Acidosis-induced activation of GPR4 caused an increase in endothelial permeability, enhanced monocyte-EC interaction and inflammatory responses, and endoplasmic reticulum stress in ECs^11–14^. Moreover, a recent study in mice has shown that GPR68 expressed in EC from small-diameter blood vessels is essential for flow-mediated dilation of mesenteric arteries^15^. Other studies suggested the role of GPR68 in intestinal inflammation, colitis, and fibrosis^16,17^. However, the direct involvement of GPR68 in regulating lung endothelial barrier function and inflammation remains unknown.

OGM-8345 is a small molecule GPR68 inhibitor recently discovered by our group via chemical genetic screening of GPCRs, and the compound was named ogremorphin owing to its capability to induce perturbed pigmentation of zebrafish embryos^18^. OGM-8345 acts as a highly specific inhibitor of GPR68, exhibiting sub-micromolar potency. It effectively inhibits GPR68 activity at concentrations as low as 0.5–2 µM in cell models of glioblastoma, where it also induces ferroptotic cell death across a variety of glioblastoma lines, including those resistant to standard chemotherapy like temozolomide^19^. The specificity of OGM-8345 was validated through screening against a broad panel of 158 GPCRs and 442 kinases. Further analyses, including genetic studies, confirmed that its effects in zebrafish models were primarily due to GPR68 inhibition. OGM-8345 did not significantly affect the activity of related proton-sensing receptors, such as GPR4 and GPR65, and was well tolerated in non-malignant cells and zebrafish, highlighting its selective inhibition of GPR68^19^.

In contrast, a small molecule Ogerin functions as a positive allosteric modulator (PAM) of GPR68, enhancing the receptor’s sensitivity to proton-mediated activation. It was identified through a virtual screening of over 3.1 million compounds and has been optimized through structure–activity relationship (SAR) studies, resulting in analogs like MS48107 that exhibit a 33-fold increase in allosteric activity compared to the original compound^20^. Ogerin selectively enhances GPR68 activity without affecting other proton-sensing GPCRs, such as GPR4 and GPR65, or a wide range of other GPCRs and drug targets^20^. Functional assays have confirmed that Ogerin potentiates GPR68-mediated Gs-cAMP signaling specifically in response to acidic conditions, making it a useful tool for studying the physiological roles of GPR68 in environments where pH is a critical factor. Together, OGM-8345 and Ogerin provide complementary approaches to modulating GPR68 activity.

In the present study, we used OGM-8345 to assess the effects of GPR68 inhibition on lipopolysaccharide (LPS)-induced dysfunction of human pulmonary EC and acute lung injury in the animal model. Furthermore, using gain or loss of function molecular approaches, we evaluated the role of GPR68 in regulation of LPS-induced EC injury.

## Materials and methods

### Reagents

GPR68 inhibitor OGM-8345 was routinely synthesized in our lab and dissolved in DMSO (10–40 mg/ml). LCMS and NMR analysis was performed for each newly synthesized batch^19^. GPR4 inhibitor NE52-QQ57 (IC_50_ = 70 nM) was purchased from MedKoo Biosciences Inc. (Morrisville, NC) and was also dissolved in DMSO. LPS (*E. coli* 0111: B4) and antibodies to VE-cadherin, and ICAM-1, were obtained from Santa Cruz Biotechnology (Santa Cruz, CA). Synthetic Lipid A analog CRX-527 was from Invivogen (San Diego, CA). IkBα, phospho- and total NF-kB, VCAM-1 and HRP-linked anti-mouse and anti-rabbit antibodies were from Cell Signaling (Beverly, MA). Ogerin was from Sigma (St. Louis, MO), and GPR68 antibody and DNA/siRNA transfections reagents were purchased from Thermo Fisher Scientific (Waltham, MA).

### Cell culture

Human pulmonary artery endothelial cells (HPAECs) obtained from Lonza (Allendale, NJ) were cultured on endothelial growth media following manufacturer’s instructions. For experiments, cells at early passages 5–8 were stimulated on media with 2% fetal bovine serum. Human lung microvascular EC and complete culture media were obtained from Promega (Heidelberg, Germany).During stimulations, control cells received the same amount of DMSO (final concentration 0.1%) as the inhibitor-treated groups.

### DNA and siRNA transfections

Plasmid DNA transfections with wild type GFP-tagged GPR68 and inactive truncated GFP-tagged GPR68 mutant (E336X-GFP) lacking 30 amino acids in C-terminus^19^ were carried out using Lipofectamine LTX with plus reagent (Thermo Fisher Scientific). After 24 h of transfections, cells were subjected to permeability and other analysis. Pre-designed non-specific or siRNA targeting GPR68 were obtained from Dharmacon (Horizon discovery ltd., Lafayette, CO) and transfections were performed using Lipofectamine RNAiMAX reagent (Thermo Fisher Scientific). All assays were done after 72 h of siRNA transfections.

### Permeability assays

Transendothelial electrical resistance (TER) reflecting EC permeability was monitored across EC monolayers in an electric cell-substrate impedance sensing system (Applied Biophysics, Troy, NY) as described in detail earlier^21^. Cells reaching > 1200 ohms of steady state resistance were subjected for TER analysis and normalized resistance were plotted against time to determine EC barrier integrity. Similarly, endothelial permeability to macromolecules was determined by express permeability testing (XPerT) assay developed by our group^22^. Images of FITC-avidin bound to the biotinylated gelatin matrix were captured using Nikon Eclipse TE 300 microscope (Nikon, Tokyo, Japan).

### Immunofluorescence microscopy

EC junctions were visualized by immunostaining with VE-cadherin antibody. Briefly, at the end of agonists stimulations, cells were fixed in 3.7% formaldehyde solution in PBS for 10 min. at 4 °C followed by permeabilization with 0.1% Triton X-100 for 30 min at room temperature and blocked with 2% BSA in PBS for 30 min. VE-cadherin antibody was added for 1 h at room temperature followed by staining with Alexa 488-conjugated secondary antibody. Texas Red-conjugated phalloidin was added to visualize actin filaments. The slides were analyzed in a Nikon Eclipse TE300 inverted microscope connected to SPOT RT monochrome digital camera and image processor (Diagnostic Instruments, Sterling Heights, MI). Adobe Photoshop 7.0 (Adobe Systems, San Jose, CA) was used to process images. The quantitative analysis of paracellular gap formation was performed as described previously^23,24^. Briefly, paracellular gaps were manually marked out on the captured images of EC monolayers and the gap formation was expressed as a ratio of the gap area to the area of whole image calculated by ImageJ software (version 1.54p, NIH, Bethesda, MD).At least 10 different microscopic fields representing both the central and peripheral areas of the plate were analyzed for each experimental condition.

### GPR68 activation assay

GPR68 activation was determined by employing PRESTO-Tango assay as described earlier^25^. Briefly, cells seeded on 6-well dishes were transfected with a combination of GPR68 Tango, β-arrestin, and Luciferase plasmids for 24 h. After desired periods of agonists stimulation, luciferase assay was carried out with Bright-Glo reagent from Promega by measuring luminescence on a VICTOR X5 microplate reader (PerkinElmer, Waltham, MA).

### Intracellular calcium measurements

Chem1-GPR68 cells were plated on μ-slide 1 Luer (Ibidi) and stained with calcium indicator dye Fura-2 according to manufacturer’s instructions (Thermo Fisher Scientific). Cells were then exposed to acidic pH-6.5 with or without LPS treatment, generating a robust rise of intracellular Ca^2+^ that could be inhibited by a 5-min pre-incubation with 3 μM OGM-8345 or 1 μM GPR4 inhibitor.

### Western blotting

Protein samples were separated on 8% SDS-PAGE and transferred onto polyvinylidene fluoride membranes using semi-dry transfer system (17 V, 1 h, Bio-Rad). Membranes were blocked in 3% BSA for 1 h at room temperature followed by incubation with desired primary antibodies at 4 °C overnight and horse radish peroxidase-conjugated secondary antibodies at room temperature for 1 h. Protein bands were detected using an enhanced chemiluminescent system (Thermo Fisher Scientific) and images densitometry was determined with Image J software (version 1.54p, NIH, Bethesda, MD).

### Quantitative real-time PCR

Total RNA extraction was done using RNeasy plus kit (Qiagen, Germantown, MD) and one microgram of cDNA was synthesized with iScript cDNA synthesis kit (Bio-Rad, Hercules, CA). The quantitative real-time PCR was performed on a Bio-Rad CFX96 real-time PCR system with SYBR green (Quantabio, Beverly, MA). Ct values were normalized to GAPDH and fold changes in gene expression was calculated using ΔΔCt method. Primers used in qPCR are listed in Supplemental data, Table S1.

#### Measurement of inflammatory markers

The secretory levels of adhesion molecule soluble ICAM (sICAM), cytokine IL-6, and chemokine IL-8 in conditioned media were determined by running ELISA with commercially available kits (R&D Systems, Minneapolis, MN). The concentration of cytokines was calculated by generating a standard curve with the absorbance readings measured at 450 nm.

#### Acute lung injury (ALI) mouse model

C57BL/6 mice were purchased from Jackson Laboratories (Bar Harbor, ME) and all the protocols for animal care and treatment were approved by the Institutional Animal Care and Use Committee (IACUC) of University of Maryland. All methods involving animals treatments were performed in accordance with the guidelines and regulations of IACUC of University of Maryland and are reported in accordance with ARRIVE guidelines. Mice were exposed to OGM-8345 (40 mg/kg, i.p.) immediately followed by LPS (0.75 mg/kg, i.t.). After 18 h, animals were sacrificed following anesthetization with ketamine (12 mg/kg, i.p.). Lung injury parameters were evaluated by determining the accumulation of Evans blue in the lung, and measurement of total cells, protein in bronchoalveolar lavage (BAL) as described previously^26,27^. The quantification of Evans blue dye accumulated in the lung tissue was done by spectrophotometric analysis of lung tissue lysates following the earlier described protocol^28^. Briefly, formaldehyde extracts of lungs were centrifuged at 12,000 × g to obtain the homogenates. Thus obtained supernatants were run for colorimetric analysis at a spectrophotometer (Molecular Devices, CA, USA) at dual wavelengths of 620 nm and 750 nm against a blank of 50% formaldehyde in PBS. qPCR was performed to determine the levels mRNA expression of selected inflammatory marker genes.

#### Statistical analysis

Results are expressed as means ± SE of three to eight independent experiments. The Unpaired Student’s *t* test was utilized to compare between control and stimulated groups, and one-way ANOVA followed by the post hoc Fisher’s test was used for comparison of multiple groups. *P* < 0.05 was considered as statistically significant.

## Results

### GPR68 is ubiquitously expressed and specifically activated by LPS in EC

**Fig. 1:**
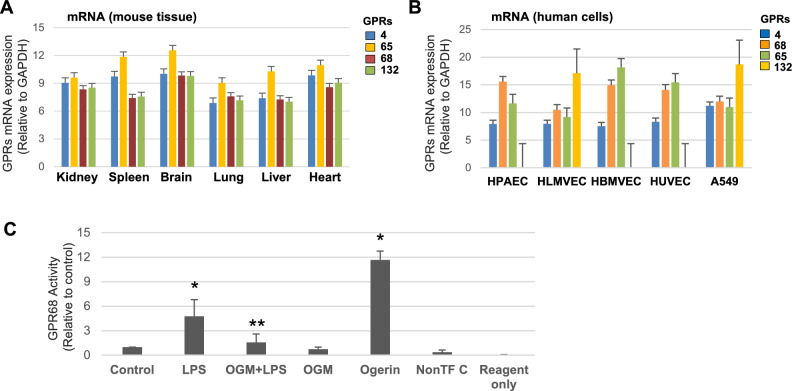
GPR68 tissue expression and activation by LPS. (**A**, **B**) Total RNA was isolated from indicated mouse tissues (**A**) or various EC subtypes (**B**), and mRNA expression of GPR4, GPR65, GPR68, and GPR132 was determined by qPCR. Relative expression levels of indicated GPCRs were obtained by calculating ΔCt values since GAPDH Ct values were largely consistent across all tissues and ECs. **(C)** HPAECs transfected with GPR68 PRESTO-Tango plasmids (24 h) were stimulated with Ogerin (10 µM, 6 h) or LPS (100 ng/ml) with or without OGM-8345 pretreatment (3 µM, 30 min). GPR68 activity was measured with luciferase assay. The results are expressed as a fold change over transfected, non-stimulated control groups. *p < 0.05, n = 6.

Among the proton-sensing GPCRs, GPR4 and GPR68 show ubiquitous expression^29^, while GPR65 is exclusively present in lymphoid tissue^30^ and GPR132 is abundantly expressed by immune cells^31^. Analysis of mRNA expression levels in mouse tissues showed the presence of all four proton-sensing GPCRs at comparable levels (Fig. 1A). Expression of these GPCRs was also evaluated in endothelial cell cultures from various vascular beds. The results demonstrated the presence of all three receptors: GPR4, GPR65, and GPR68 in lung EC of macro- and microvascular origin, brain microvascular, and umbilical cord vein EC, as well as in human lung epithelial cell line A549. In contrast, GPR132 was not detected in macrovascular EC (Fig. 1B).

Functional activation of GPR68 receptor by LPS was further assessed by PRESTO-Tango assay described in Methods. A selective positive allosteric modulator of GPR68 Ogerin was used as a positive control. LPS treatment led to a nearly five-fold increase in GPR68 activity. Importantly, EC treatment with GPR68 inhibitor OGM-8345 abolished LPS-induced GPR68 activation (Fig. 1C).

### OGM-8345 inhibits LPS-induced GPR68 activation and calcium flux

Using chemical genetic screening of zebrafish embryonic development, our group has previously identified a small molecule OGM-8345 (Fig. S1A) as a potent GPR68-specific inhibitor (IC_50_ = 0.71 µM)^18^. The coupling of GPR68 to Gα_q/11_ activates phospholipase C leading to the release of intracellular calcium^29^. In addition to PRESTO-Tango GPR68 activation assay, we evaluated LPS-induced activation of GPR68 by measuring intracellular Ca^2+^ levels in control and LPS-challenged human pulmonary EC in the presence and absence of GPR68- and GPR4-specific inhibitors. There was a significant increase in intracellular Ca^2+^ levels in LPS-treated group as compared to control and more importantly, incubation with OGM-8345 completely abolished the intracellular Ca^2+^ release induced by LPS (Fig. S1B). In contrast, a selective GPR4 antagonist NE52-QQ57 did not exhibit any inhibitory effects on LPS- induced intracellular Ca^2+^ release in EC (Fig. S1B). EC incubation with OGM-8345 did not affect mRNA levels of GPR4, GPR65 and GPR68 (Fig. S1C).

### OGM-8345 attenuates LPS-induced EC barrier disruption

**Fig. 2:**
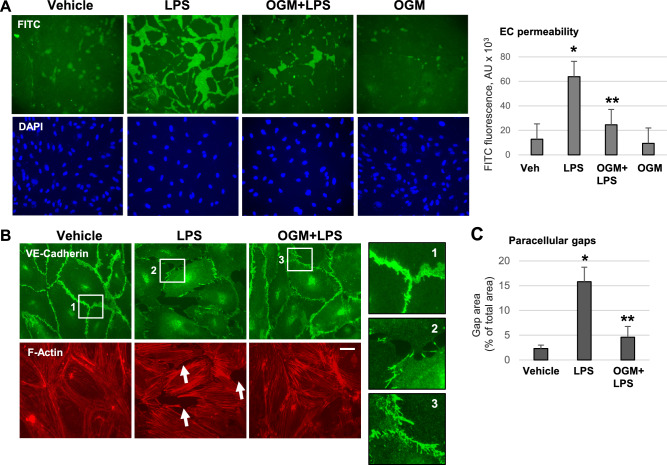
OGM protects against LPS-induced endothelial barrier disruption in lung EC. (**A**) HPAECs were pre-incubated with OGM-8345 (3 µM, 30 min) followed by stimulation with LPS (100 ng/ml, 6 h). XPerT assay was performed to evaluate endothelial permeability as described in the Methods section. Cell nuclei were stained with DAPI. Increased FITC fluorescence reflects endothelial macromolecular permeability. *p < 0.05, versus control and **p < 0.05, versus LPS, n = 4. (**B**) Cells were subjected to similar treatments as in (**A**); F-actin and VE-cadherin staining was carried out to visualize actin filaments and EC junctions correspondingly. Insets: high magnification images of adherens junctions. Bar = 5 µm. Arrows indicate paracellular gap formation. Quantification analysis is shown in (**C**). *p < 0.05, versus control and **p < 0.05, versus LPS, n = 6.

The physiological significance of OGM-8345-mediated inhibition of GPR68 activation caused by LPS was evaluated in experiments testing effects of OGM-8345 on EC permeability caused by inflammatory agonists. EC monolayer permeability for macromolecules was evaluated by XPerT visualization assay described in Methods. LPS doses were optimized for each assay to better demonstrate cell responses to treatments. Initial tests established submaximal inflammatory response to 25 ng/ml, maximal inflammatory effect at 50 ng/ml and maximal effect on EC permeability and cytoskeletal remodeling at 100 ng/ml LPS dose. No cell death was observed even with highest dose of LPS used as verified by cell viability assay performed up to 72 h of LPS stimulations. Treatment of HPAEC monolayers with LPS disrupted EC monolayer integrity leading to accumulation of a tracer, FITC-labeled avidin, on biotinylated substrate underneath the EC monolayers (Fig. 2A). Cell pretreatment with OGM-8345, while not affecting EC barrier in control conditions, strongly attenuated EC hyperpermeability caused by LPS. In parallel, EC barrier breach caused by LPS was also monitored by immunostaining of adherens junctions with VE-cadherin antibody or Texas-Red Phalloidin to visualize F-actin filaments. LPS exposure caused discontinuous patterns of VE-cadherin staining at cell junctions reflecting loss of adherens junction integrity and breakdown of EC barrier (Fig. 2B, upper panels and insets). Pre-incubation with OGM-8345 prevented LPS-induced partial disassembly of adherens junctions illustrated by preserved continuous pattern of peripheral VE-cadherin staining. OGM-8345 also attenuated remodeling of the actin cytoskeleton in response to LPS and suppressed formation of paracellular gaps as evidenced by staining of F-actin (Fig. 2B, lower panels). Quantification of LPS-induced gap formation and effects of OGM-8345 is depicted in Fig. 2C.

### OGM-8345 suppresses agonist-induced EC inflammation

**Fig. 3:**
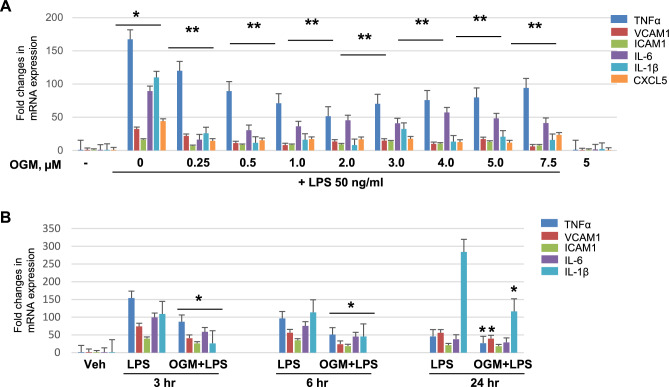
OGM attenuates LPS-induced inflammation in lung EC. (**A**) HPAECs were exposed to indicated concentrations of OGM-8345 for 30 min prior to stimulation with 50 ng/ml LPS for 3 h. qPCR was performed to measure mRNA levels of endothelial pro-inflammatory marker genes TNF-α, VCAM-1, ICAM-1, IL-6, IL-1β, and CXCL5. *p < 0.05, versus control and **p < 0.05, versus LPS, n = 4. (**B**) Cells were pre-treated with OGM-8345 (3 µM, 30 min) followed by stimulation with 50 ng/ml of LPS for indicated time periods. mRNA expression of selected inflammatory genes was determined by qPCR. *p < 0.05, vs. LPS, n = 3.

Anti-inflammatory effects of OGM-8345 were evaluated in the next experiments. RT-PCR analysis showed that OGM-8345 attenuated LPS-induced mRNA expression of EC inflammatory markers: tumor necrosis factor-α (TNF-α), intercellular adhesion molecule-1 (ICAM-1), vascular cell adhesion molecule-1 (VCAM-1), interleukins IL-6, IL-1β, and C-X-C motif chemokine ligand 5 (CXCL5) in a dose-dependent manner with optimal concentration range of 1–3 µM (Fig. 3A and S2A). Of note, EC treatment with OGM-8345 alone did not affect mRNA expression of inflammatory markers (Fig. S2B). OGM-8345 exhibited sustained anti-inflammatory effect which was observed over 24-h period of LPS exposure (Fig. 3B).

Among the Toll-like receptors (TLR) family members, TLR4 is activated by bacterial LPS and TLR4 signaling serves as the major pathway during LPS-induced inflammatory responses in lung endothelium. CRX-527, a synthetic lipid A mimic, is a highly specific and potent TLR4 agonist. It was utilized in this study as a TLR4 activator to further verify the results obtained with LPS. OGM-8345 also inhibited EC permeability (Fig. S3A), transcriptional activation of inflammatory markers (Fig. S3B) and expression of EC adhesive proteins ICAM1 and VCAM1 (Fig. S3C) induced by CRX-527.

**Fig. 4:**
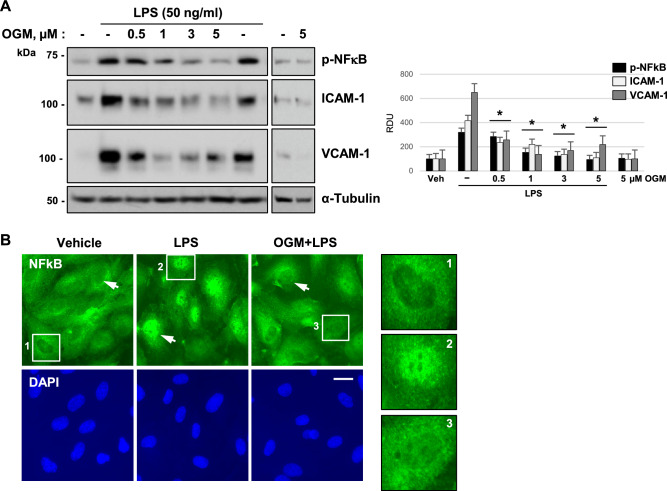
GPR68 inhibition rescues LPS-induced endothelial inflammatory activation. (**A**) HPAECs were incubated with indicated concentrations of OGM-8345 for 30 min followed by addition of LPS for 6 h. Protein levels of phospho-NFkB, VCAM-1, and ICAM-1 in cell lysates were determined by western blotting. Probing with α-tubulin was used as a loading control. Western blot densitometry is presented in the right panel. *p < 0.05, versus LPS, n = 3. **(B)** Cells were pretreated with OGM-8345 (3 µM, 30 min) following LPS challenge (50 ng/ml, 6 h). Immunofluorescence staining was carried out with NFkB antibody. Cell nuclei were stained with DAPI. Bar = 10 µm. Insets: NF-kB accumulation in nuclei. Arrows indicate nuclear translocation of NFkB. Shown are representative results of 5 independent experiments.

The inhibition of endothelial inflammation by OGM-8345 was further confirmed by analysis of NF-kB pathway activation performed by western blot and immunofluorescence staining. LPS-induced activation of canonical NF-kB pathway is characterized by increased levels of phospho-NF-kB. This effect was attenuated by preincubation with OGM-8345. Likewise, OGM-8345 suppressed LPS-induced expression of EC adhesion molecules ICAM-1 and VCAM-1 (Fig. 4A). EC treatment with OGM-8345 alone had no effect of NFkB phosphorylation and ICAM-1 and VCAM-1 expression. Nuclear translocation of NF-kB is the next step of NF-kB signaling pathway. Immunofluorescence staining with corresponding antibody demonstrated NF-kB redistribution from diffuse cytoplasmic staining to nucleus-specific accumulation; this effect was also attenuated by OGM-8345 (Fig. 4B and insets).

### GPR68 modulates LPS-induced endothelial inflammation

**Fig. 5:**
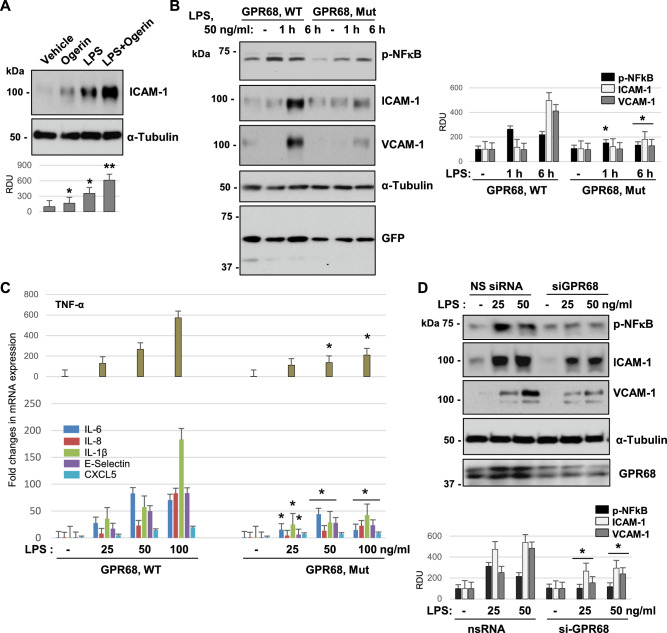
GPR68 activation or overexpression exacerbates endothelial inflammation. (**A**) Cells were treated with either 25 ng/ml LPS, 10 µM Ogerin, or both for 6 h followed by western blot analysis to detect ICAM-1 protein levels. *p < 0.05, vs. control and **p < 0.05, versus LPS, n = 3. (**B**) Cells were transfected with wild type (WT) or inactive mutant (Mut) GPR68 plasmids for 24 h followed by stimulation with 50 ng/ml of LPS for 1 or 6 h. Protein expression of phospho-NFkB, VCAM-1, and ICAM-1 were analyzed by western blotting. Probing for GFP was used as a transfection control. *p < 0.05, versus WT, n = 3 (**C**) Cells were transfected with empty vector (EV) or GPR68 wild type (WT) plasmids for 24 h and exposed to 50 ng/ml of LPS for 3 h. qPCR was performed to determine mRNA expression levels of TNF-α (upper panel), IL-6, IL-1β (middle panel), and IL-8, E-selectin (lower panel). *p < 0.05, versus control and **p < 0.05, WT versus EV, n = 3. (**C**) Cells were transfected with GPR68 WT or inactive mutant (GPR Mut) plasmids for 24 h followed by stimulation with indicated dosed of LPS (3 h). mRNA levels of TNF-α (upper panel) or IL-6, IL-8, IL-1β, E-selectin, and CXCL5 (lower panel) were determined by real-time PCR. *p < 0.05, versus WT, n = 4. **(E)** Cells were subjected to non-specific (NS) or GPR68-targeting (siGPR68) siRNA transfection for 72 h and then stimulated with LPS for 6 h. Western blot analysis was carried out to measure the protein levels of phospho-NFkB, VCAM-1, and ICAM-1. Efficiency of siRNA-mediated knockdown was verified by probing the blots with GPR68; β-actin was used as a loading control. Shown are representative results of 5 independent experiments.

The role of GPR68 in EC inflammation was investigated in the following experiments. Lower LPS dose (25 ng/ml) causing sub-maximal effect on inflammation was used in these experiments to show additive effect of Ogerin, a GPR68-specific positive allosteric modulator^20^. EC treatment with Ogerin caused modest increase in ICAM-1 expression as compared to effect by LPS (Fig. 5A). However, Ogerin strongly augmented the pro-inflammatory effect of LPS. Importantly, LPS-triggered ICAM1 induction was lost in EC expressing functionally inactive GPR68 mutant^19^ (Fig. 5B). RT-PCR analysis further confirmed that ectopic expression of GPR68 mutant diminished mRNA transcripts of endothelial inflammatory markers: TNFα, IL-6, IL-1β, IL-8, E-selectin, and CXCL-5 caused by LPS (Fig. 5C). Next, we evaluated the effects of siRNA-induced GPR68 knockdown on LPS-induced EC inflammation. Depletion of endogenous GPR68 attenuated the levels of phospho-NFkB, ICAM-1and VCAM-1 protein expression which were tested in EC challenged with two different doses of LPS (Fig. 5D). Of note, GPR68 knockdown did not affect mRNA levels of other GPRs (data not shown).

### GPR4 is not involved in LPS-induced endothelial injury

**Fig. 6:**
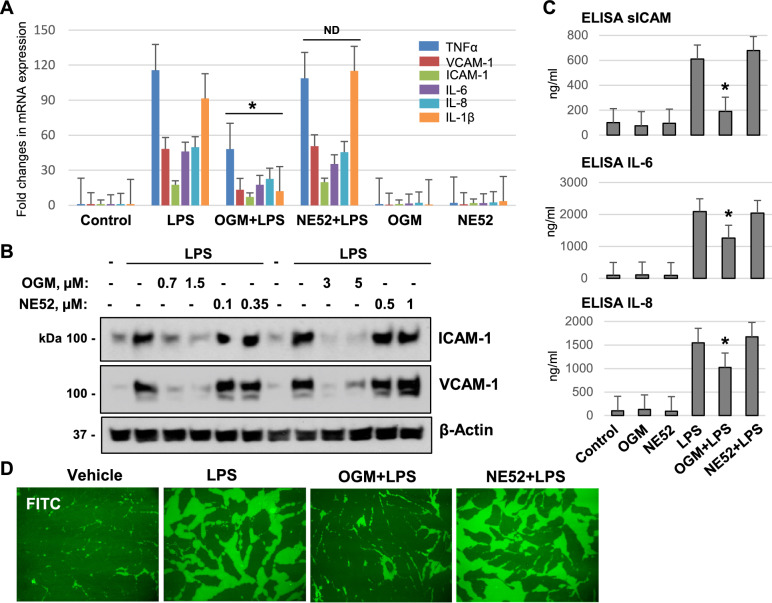
Inhibition of GPR68 but not GPR4 mitigates LPS-induced inflammation in lung EC. (**A**) HPAECs were pre-treated with 3 µM of OGM-8345 or 1 µM of NE52-QQ57 for 30 min followed by LPS stimulation (50 ng/ml, 3 h). qPCR was performed to determine mRNA expression levels of inflammatory markers. (**B**) Cells were incubated with indicated doses of GPR68 and GPR4 inhibitors (30 min) followed by LPS addition (50 ng/ml, 6 h). Protein levels of phospho-NFkB, VCAM-1, and ICAM-1 were determined by western blotting. (**C**) Cells were exposed to 3 µM of OGM-8345 or 1 µM of NE52-QQ57 followed by stimulation with LPS (50 ng/ml, 6 h). Cell media were collected for ELISA assays to detect secretory levels of sICAM1, IL-6, and IL-8. *p < 0.05, versus LPS, n = 3. (**D**) Cells were subjected to identical treatment scheme as in (**C**), and EC macromolecular permeability was determined by XPerT assay. Shown are representative FITC fluorescence images from 5 independent experiments.

Previous reports described that acidosis-activated GPR4 mediates endothelial permeability and inflammation in EC^13,14^. To determine if GPR4 is involved in LPS-induced EC dysfunction in our experimental settings, we investigated the effects of NE52-QQ57, a pharmacological inhibitor specifically targeting GPR4, on LPS-induced EC inflammatory response. GPR4 inhibition did not affect the LPS-induced increase in mRNA expression of TNF-α, VCAM-1, ICAM-1, IL-6, IL-8, and IL-1β (Fig. 6A). GPR4 inhibition did not affect the LPS-induced increase in mRNA expression of TNF-α, VCAM-1, ICAM-1, IL-6, IL-8, and IL-1β (Fig. 6A). Similar results were obtained with western blot analysis of whole cell lysates, which showed that LPS-induced expression of ICAM-1 and VCAM-1 was significantly attenuated by OGM-8345, but not affected by four different doses of NE52-QQ57 (Fig. 6B). Analysis of cytokine production by ELISA assays demonstrated that LPS-induced increase of soluble ICAM-1, IL-6, and IL-8 in EC conditioned medium was strongly inhibited by OGM-8345, but not by NE52-QQ57 (Fig. 6C). In agreement with attenuation of LPS-induced inflammatory activation, OGM-8345, but not NE52-QQ57 strongly attenuated LPS-induced EC permeability which was evaluated by XPerT permeability visualization assay (Fig. 6D).

### OGM-8345 post-treatment recovers endothelial function after LPS

**Fig. 7:**
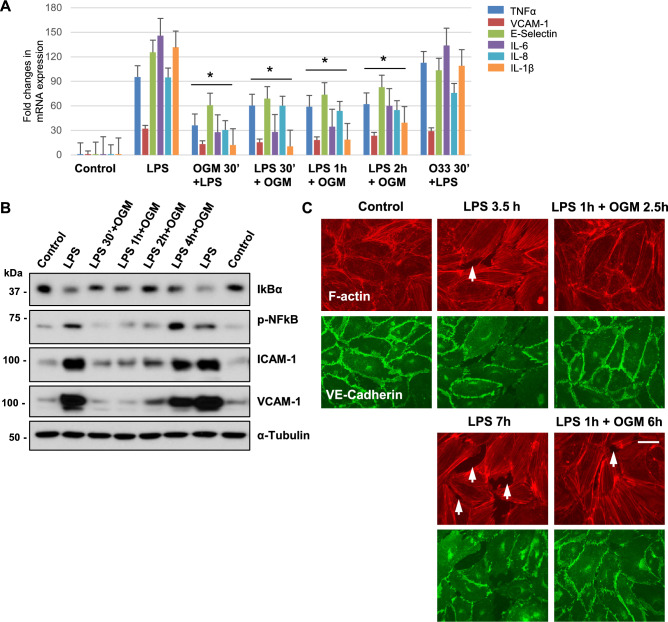
OGM post-treatment rescues LPS-induced endothelial dysfunction. (**A**) HPAECs were either pre-treated with OGM-8345 (3 µM, 30 min), or inhibitor was added at indicated times after LPS stimulation (50 ng/ml). O33 (5 µM), a non-functional structural analogue of OGM-8345 was used as a negative control. After 3-h incubation with LPS and inhibitors, qPCR analysis was conducted to determine the gene expression of selected pro-inflammatory markers. *p < 0.05, vs. LPS, n = 3. (**B**) Cells were exposed to 50 ng/ml of LPS followed by addition of 3 µM of OGM-8345 at indicated time periods. At the end of total 6 h incubation with LPS and OGM, protein levels of IkBα, phospho-NFkB, VCAM-1, and ICAM-1 were determined by western blotting; α-tubulin was used as a loading control. (**C**) Cells pre-activated with LPS (50 ng/ml, 1 h) were exposed to OGM-8345 and further incubated for 3.5 h (upper panels) or 7 h (lower panels) total. Actin cytoskeleton and adherence junction remodeling were evaluated by immunofluorescence co-staining with Texas-Red Phalloidin and VE-cadherin antibody, respectively. Shown are representative images of 3 independent experiments.

Substantial protective effects of OGM-8345 described above were achieved with 30 min EC pre-incubation with the inhibitor prior to LPS challenge. We next tested the efficacy of GPR68 inhibition in more clinically relevant settings of pre-existing pathological insult. ECs stimulated with LPS were post-treated with OGM-8345 after 30 min, 1 h, and 2 h of LPS administration. qRT-PCR data showed that LPS-induced increase in TNF-α, VCAM-1, E-selectin, IL-6, IL-8, and IL-1β mRNA expression was suppressed by OGM-8345 post-treatment 30 min, 1 h, and 2 h after LPS challenge (Fig. 7A). The data showed that inhibitory effects of OGM-8345 2-h post-treatment were comparable to that of 30-min pre-treatment. Of note, OGM-33, a structural analogue of OGM-8345 but devoid of GPR68 inhibitory activity, had no effect on LPS-induced inflammation (Fig. 7A). Protein expression analysis further confirmed that OGM-8345 protects against LPS-induced activation of NF-kB pathway detected by IkBα degradation and NF-kB phosphorylation, and increased expression of ICAM-1 and VCAM-1 within 2-h window of post-treatment, but such protective effect of inhibitor was diminished at 4-h post-treatment (Fig. 7B). Analysis of EC intercellular gap formation (Fig. 7C, upper panels) and adherence junction disassembly (Fig. 7C, lower panels) by LPS exposure demonstrated sustained, up to 7 h, preservation of EC monolayer integrity when OGM-8345 was added 1 h post-LPS (Fig. 7C).

ECs from various vascular beds are known to exhibit distinct functional characteristics including their responses to agonists^32^. To explore the broad specificity of the GPR68 inhibitor across various vascular beds, we analyzed the effects of OGM-8345 on human lung microvascular EC function. As in pulmonary EC from macrovascular bed, treatment of human lung microvascular EC with OGM-8345 attenuated LPS-induced disruption of monolayer integrity demonstrated by disappearance of VE-cadherin positive adherens junctions, actin cytoskeletal remodeling, and formation of intercellular gaps (Fig. S4A). To assess effects of GPR68 activator Ogerin on EC monolayer disruption, a lower dose of LPS was used (25 ng/ml). Similar to macrovascular lung EC, Ogerin further escalated LPS-induced disruption of monolayer integrity (Fig. S4B) and increased permeability in human lung microvascular EC (Fig. S4C). Importantly, LPS-induced EC monolayer disruption and hyperpermeability was suppressed by GPR68 inhibition with OGM-8345 (Fig. S4A,C). Also, LPS-induced activation of inflammatory cascades was substantially blocked by OGM-8345 in the microvascular EC, as demonstrated by decreased mRNA expression of VCAM-1, ICAM-1, IL-8 (Fig. S4D), and by decreased protein levels of ICAM-1 and VCAM-1 (Fig. S4E). In contrast, the inhibition of GPR4 activity by NE52-QQ57 was without effect (Fig. S4C–E).

### OGM-8345 ameliorates LPS-induced ALI in mice

**Fig. 8:**
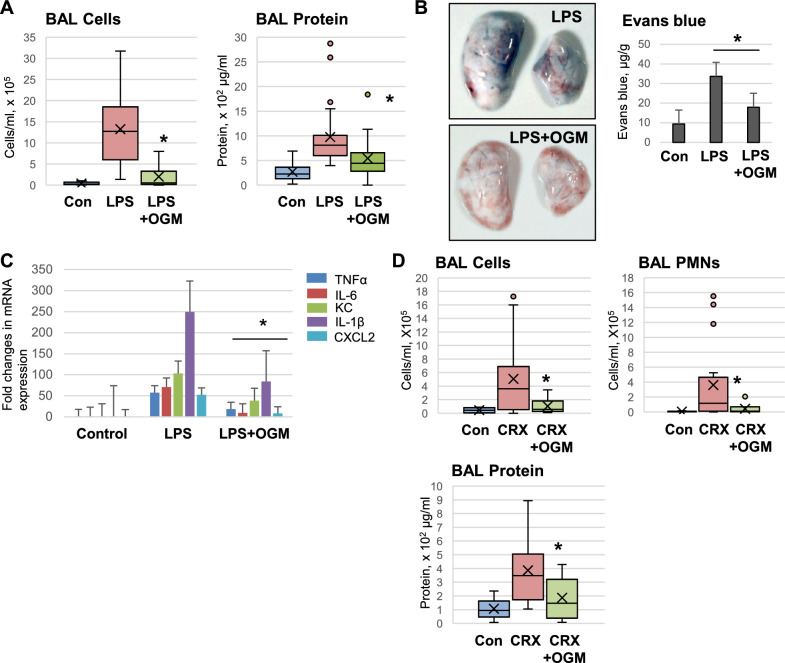
OGM ameliorates LPS-induced ALI in mice. C57/BL6J mice were injected with 40 mg/kg of OGM-8345 (i.p.) immediately followed by LPS administration (0.75 mg/kg, i.t.) (**A**) After 18 h, BAL was collected to analyze protein and cells content. (**B**) Evans blue dye (30 mg/kg, i.v.) was injected into mice 2 h prior to the end of the experiment and lung vascular permeability was assessed by visualizing Evans blue accumulation in the lung parenchyma. The quantification of Evans blue dye in the lung tissue as described in Methods is plotted on the right panel. (**C**) Total RNA was extracted from mouse lung tissues, and mRNA expression levels of TNF-α, IL-6, KC, IL-1β, and CXCL2 were determined by qRT-PCR. (**D**) C57/BL6J mice were co-treated with OGM-8345 (40 mg/kg, i.p.) and synthetic TLR4 agonist CRX-527 (1 mg/kg, i.t.) for 18 h. Total number of cells, PMNs, and protein content in BAL fluid were determined; *p < 0.05, versus LPS or CRX-527 alone, n = 8.

Protective effects of OGM-8345 were validated in the murine model of LPS-induced lung injury, as described in Methods. Mice were exposed to OGM-8345 (40 mg/kg, i.p.) immediately followed by LPS (0.75 mg/kg, i.t.). After 18 h BAL was collected to assess lung injury parameters such as total cells count and protein content. OGM-8345 treatment substantially reduced the LPS-induced increase of total cell count and protein content in BAL (Fig. 8A). Lung vascular permeability in mice was further monitored by Evans blue extravasation assay described in Methods. The images of flushed lungs from mice treated with LPS or OGM-8345 + LPS show strong attenuation of the Evans blue tracer accumulation in the lung parenchyma of OGM-8345-treated group (Fig. 8B). Lung tissues were also subjected to qRT-PCR analysis, and the results showed that OGM-8345 treatment led to a significant reduction of lung inflammation reflected by a decrease of LPS-induced mRNA transcripts: TNF-α, IL-6, KC, IL-1β, and CXCL2 (Fig. 8C). Similar protective effects of OGM-8345 against accumulation of protein and inflammatory cells in BAL fluid were observed in the mouse model of lung injury caused TLR4 agonist CRX-527 (Fig. 8D).

## Discussion

The present study reports for the first time the role of GPR68 in propagation of endothelial dysfunction and lung injury caused by bacteria-derived pathogens. The results also show direct involvement of GPR68 in modulation of innate immune response. These conclusions are supported by the following major findings from this study : a) inflammatory agonist LPS caused significant activation of GPR68 which was suppressed by a novel GPR68 small molecule inhibitor OGM-8345, b) Functionally, OGM-8345 exhibited protective effects against LPS-induced EC barrier compromise in lung macro- and micro-vascular EC, yet pharmacological inhibitor of GPR4 was without effect, c) GPR68 inhibitor was protective against LPS-induced vascular leak in the lung and exerted potent anti-inflammatory activities in vivo, and d) loss-of-function molecular approaches using ectopic expression of inactive GPR68 mutant or silencing of GPR68 in lung EC confirmed results of pharmacological inhibition of GPR68 activity using first-in-class small molecule inhibitor OGM-8345. GPR68 is known to be involved in multiple essential physiological functions including vascular homeostasis, neuroplasticity, intestinal inflammation, and cancer progression^33^, and our results expand its functional role as regulator of vascular endothelial permeability and inflammation.

Encouraged by a recent study reporting the essential role of GPR68 in vascular endothelial physiology and control of vasomotor tone^15^, we explored a different modality of GPR68 in potential control of EC barrier and inflammatory status in the models of lung injury. OGM-8345 blocking effect on rapid intracellular Ca^2+^ influx by LPS-stimulated pulmonary EC may be a part of protective mechanism, as blocking Ca^2+^ in LPS model attenuated EC dysfunction [Reviewed in^34^. These findings are consistent with an earlier study showing the requirement of GPR68 in flow-induced elevation of intracellular Ca^2+^ in EC^15^. GPR68-mediated increase in intracellular Ca^2+^ concentrations has been associated with activation of Gq-phospholipase C signaling axis^29,35^. Additionally, GPR68 activation has been shown to mediate the production of cAMP and prostaglandin I_2_ in human aortic smooth cells^36^. Notably, GPR68 has been described to act as a mechanosensor responding to substrate stiffness^37^, and also recently reported to be involved in histamine-induced secretion of Von Willebrand Factor by EC under shear stress^38^. Thus, elucidation of precise molecular mechanism underlying EC-protective effects of OGM-8345 warrants further investigation.

One striking observation of this study was a direct functional activation of GPR68 in lung EC in response to LPS stimulation. In line with our findings, LPS, TNF-α, and phorbol myristate acetate also induced the expression of GPR68 in human and murine monocytes^39^. Taken together, these findings provide compelling evidence of GPR68 as a sole proton-sensing GPCR responsible for mediating inflammatory response at least in lung EC. We also acknowledge involvement of another receptor, GPR4, in endothelial dysfunction caused by medium acidosis^11,13,14^, while our data indicate that GPR4 was not involved in EC permeability and inflammation caused by LPS. We also cannot exclude other factors such as EC heterogeneity or specific experimental conditions are also contributing factors. However, the involvement of other proton-sensing GPCRs cannot be completely ruled out.

Another major focus of this study was to evaluate the effects of the new GPR68 inhibitor on pulmonary EC dysfunction and ALI caused by endotoxin LPS in more clinically relevant scenario where therapeutic interventions are performed for pre-existing disease state. The results showed effectiveness of OGM-8345 post-treatment within 2-h therapeutic window after inflammatory insult, thus bolstering its therapeutic potential. OGM-8345 endothelial-protective effect encompassed suppression of a broad range of LPS-induced inflammatory markers including NF-kB pathway, TNF-α, VCAM-1, ICAM-1, IL-6, IL-8, IL-1β, IL-1α, CXCL5, and E-selectin. In support of our data demonstrating crucial role of GPR68 in inflammatory activation of pulmonary EC, earlier studies demonstrated NF-kB dependent production of IL-8 in human pancreatic β cells via GPR68 activation^40^ and IL-6 production in human aortic smooth muscle cells^41^. GPR68 inhibitor was shown to decrease TNF-α and IL-6 mRNA levels in colon tissues during dextran sulfate sodium-induced colitis^16^. A cooperation between GPR68 expression and NF-kB activation was further evidenced during hypoxia-induced upregulation of GPR68 in various immune cells^42^ and acidic pH-induced NF-kB-dependent production of CXCL8 in human aortic smooth muscle cells^43^. Of note, although potent inhibition of LPS-induced endothelial dysfunction in a therapeutic model where the compound was administered 2 h post-insult suggests its therapeutic potential, lack of testing its efficacy at much later time points of injury represents the limitations of the present study. Likewise, LPS-induced ALI does not truly mimic clinical ARDS, thus future studies are required to test the therapeutic potential of this inhibitor in more clinically relevant models such as using live bacteria-induced ALI. Furthermore, the impact of GPR68 inhibition on the overall host defense cannot be ignored and needs to be carefully evaluated considering the reported role of acidification in superoxide production^44^ and neutrophil infiltration^40^.

The urgent need for therapeutics for endothelial dysfunction-derived severe respiratory illnesses such as ARDS is further emphasized by the pandemic COVID-19 which has already taken over 6 million human lives globally. We believe that GPCRs represent most attractive therapeutic intervention since existing data suggest that approximately 34% of FDA approved drugs primarily target about 13% of all known GPCRs^45^. In this context, the GPR68 specific small molecule inhibitor OGM-8345 described in this study holds promise to be developed into a plausible drug candidate for therapeutic treatment of lung inflammatory syndromes associated with endothelial dysfunction owing to its pronounced EC barrier protective and anti-inflammatory properties.

## Supplementary Information

Supplementary Information 1.

Supplementary Information 2.

Supplementary Information 3.

Supplementary Information 4.

Supplementary Information 5.

Supplementary Information 6.

Supplementary Information 7.

Supplementary Information 8.

Supplementary Information 9.

Supplementary Information 10.

Supplementary Information 11.

Supplementary Information 12.

Supplementary Information 13.

Supplementary Information 14.

## Data Availability

All data generated or analyzed during the current study are included in the published article and its supplementary information files.
