# Supplementary figures and images for: Novel small molecule inhibitor of GPR68 attenuates endothelial dysfunction and lung injury caused by bacterial lipopolysaccharide
Source: Sci Rep. 2025 Nov 5;15:38669. doi: 10.1038/s41598-025-02582-y (PMC12589648; doi:10.1038/s41598-025-02582-y)

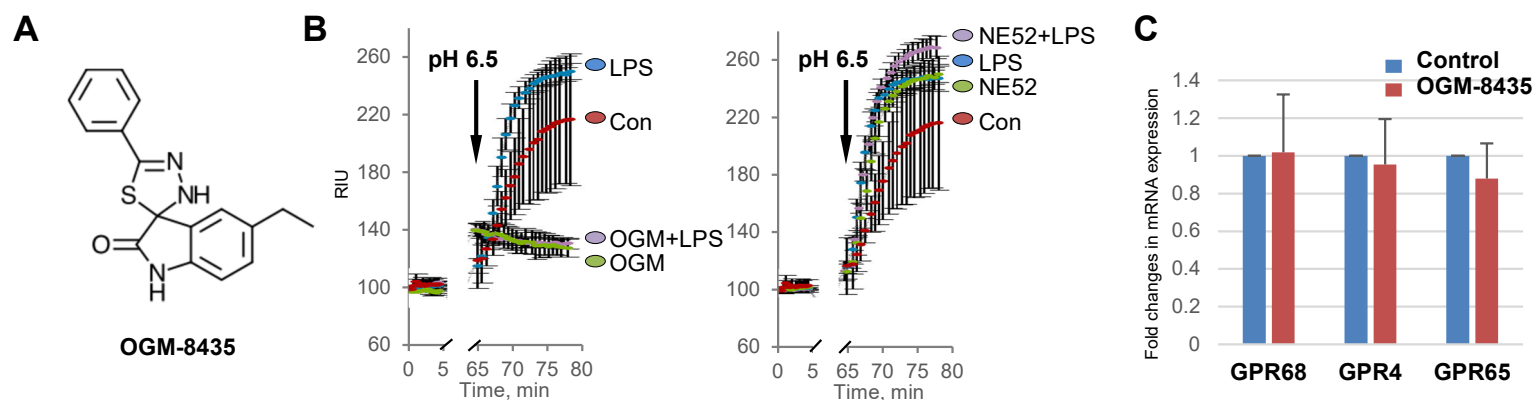

Supplement: Supplementary file 1 — Supplementary Information 1. [file 41598_2025_2582_MOESM1_ESM.pdf]

**A**

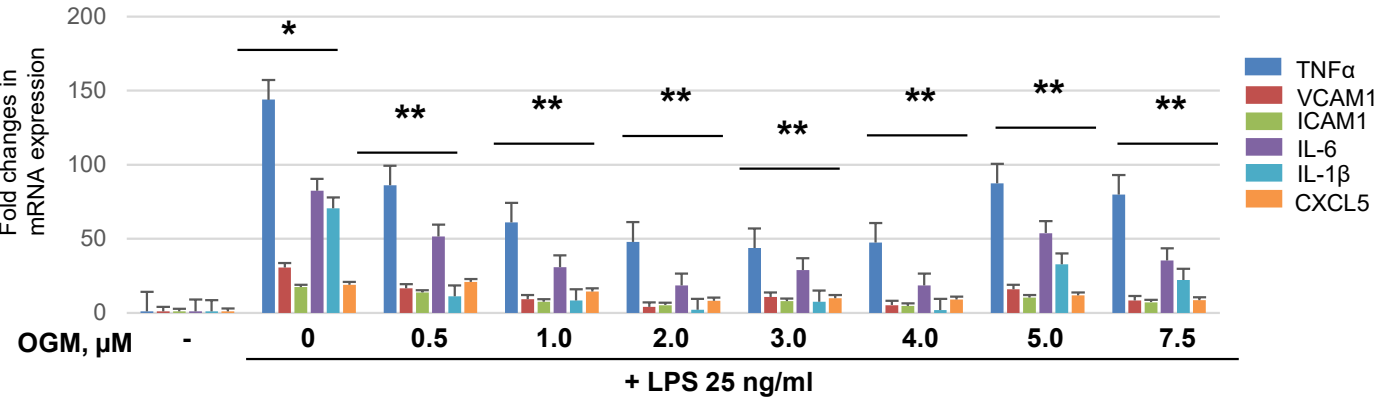

**B**

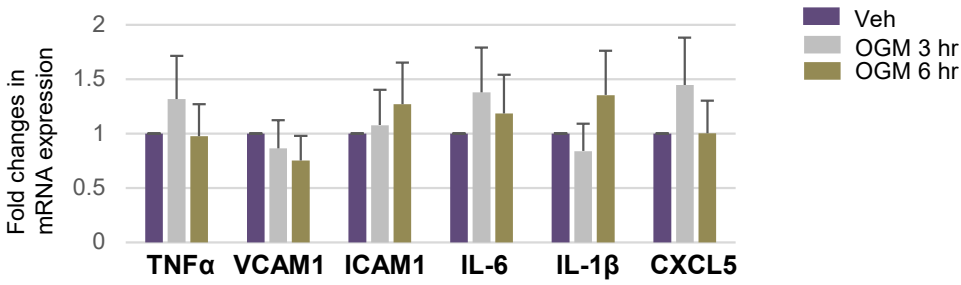

Supplement: Supplementary file 2 — Supplementary Information 2. [file 41598_2025_2582_MOESM2_ESM.pdf]

**A**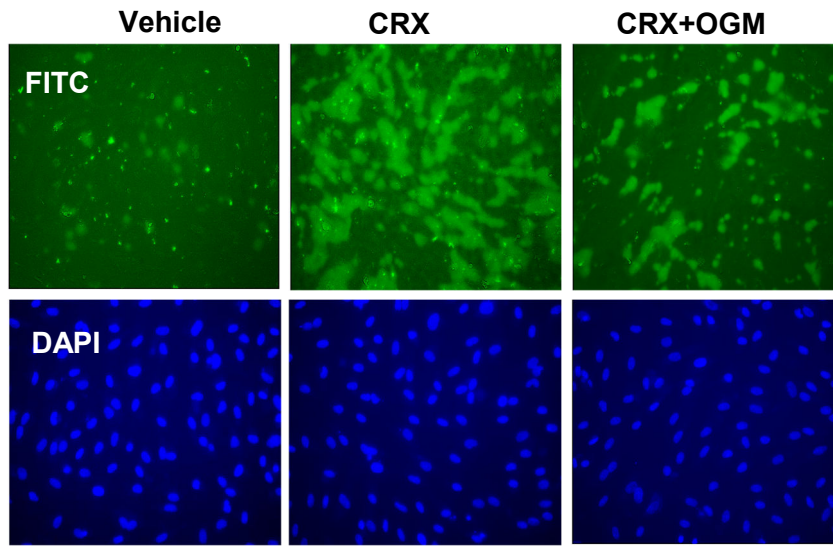**B**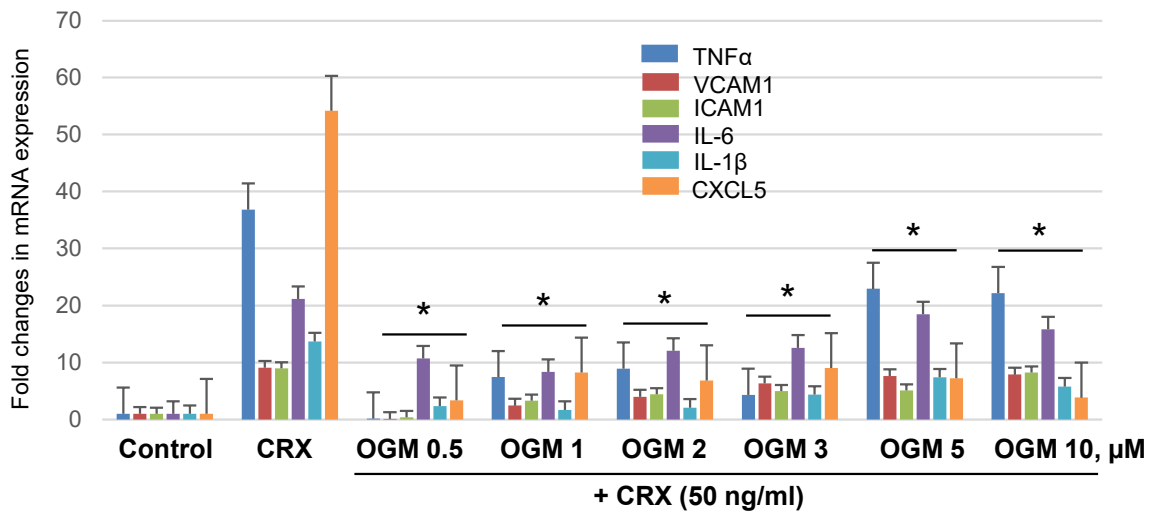**C**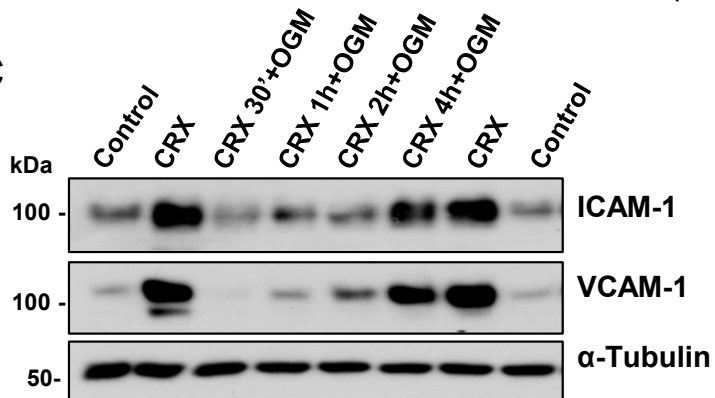

Supplement: Supplementary file 3 — Supplementary Information 3. [file 41598_2025_2582_MOESM3_ESM.pdf]

**A**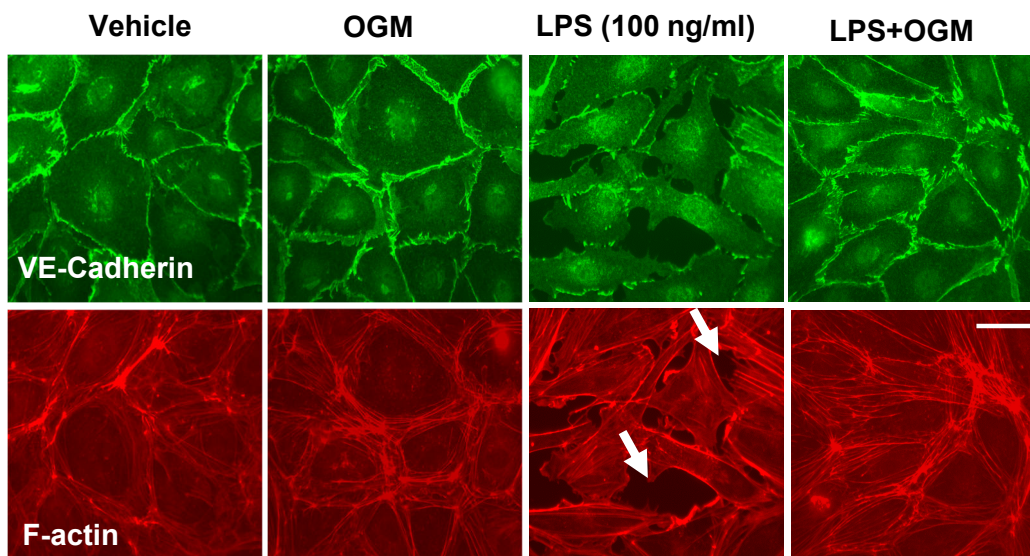**B**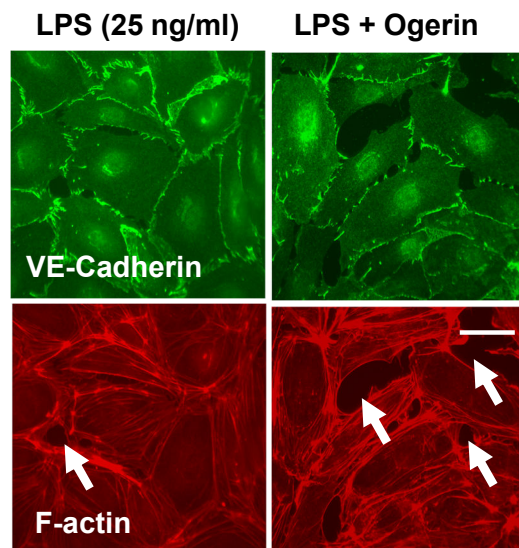**C**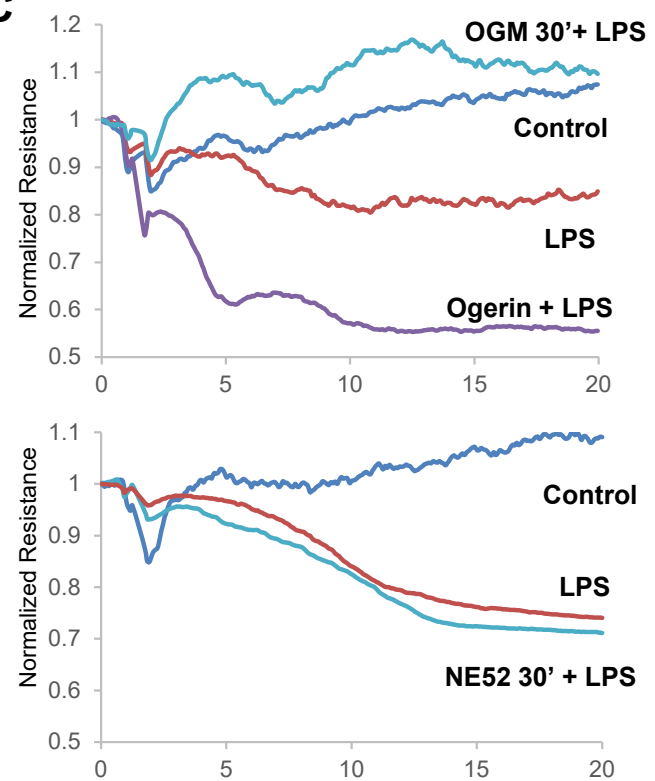**D**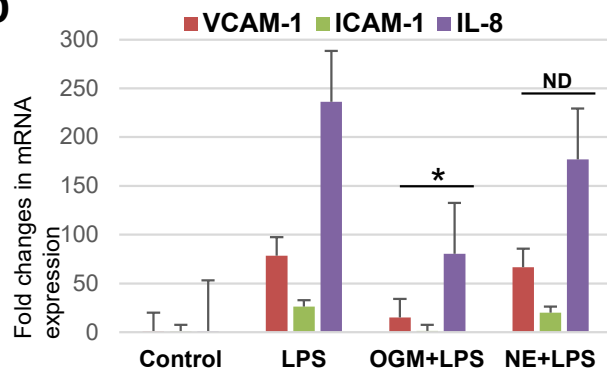**E**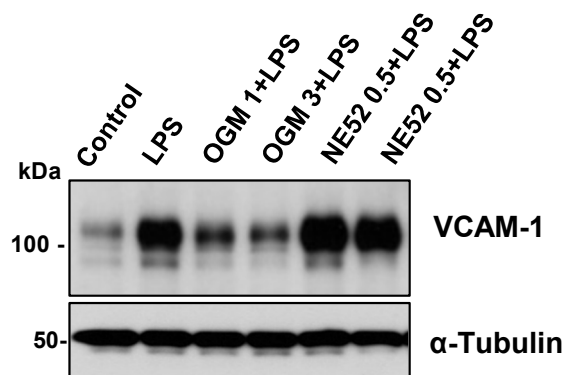

Supplement: Supplementary file 4 — Supplementary Information 4. [file 41598_2025_2582_MOESM4_ESM.pdf]

**Figure 5E**

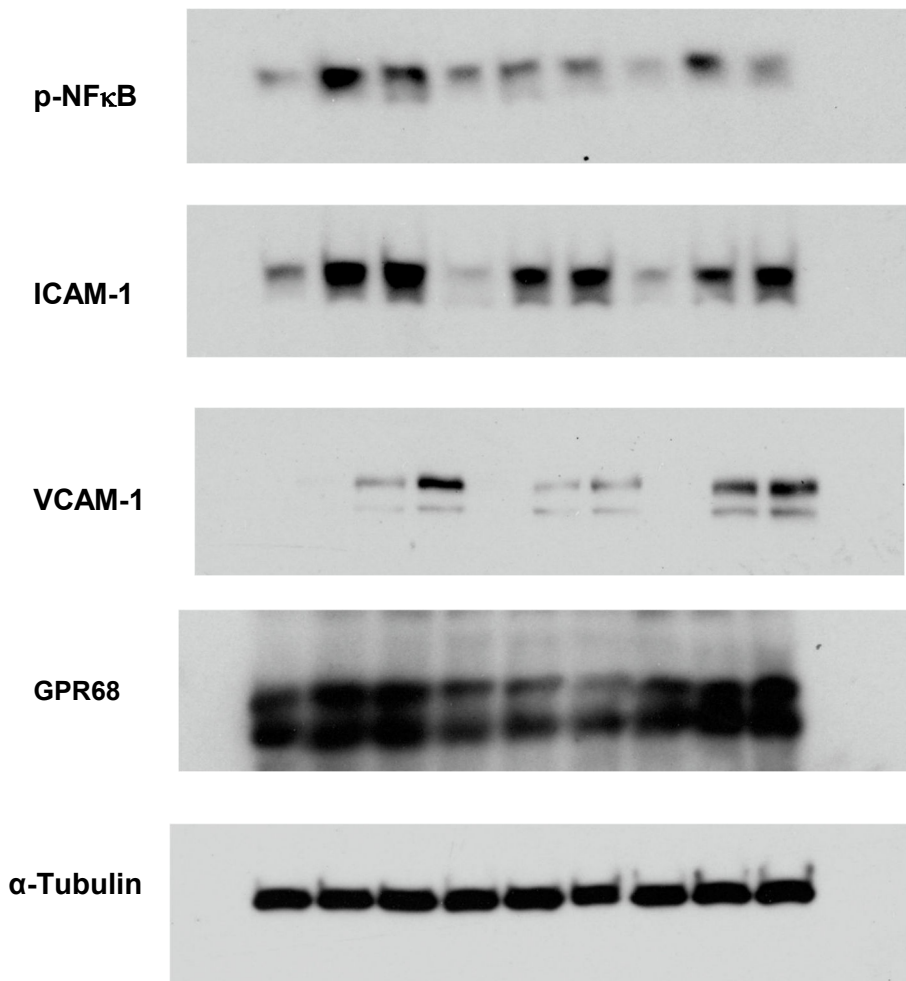

Supplement: Supplementary file 6 — Supplementary Information 6. [file 41598_2025_2582_MOESM6_ESM.pdf]

**Figure 4A-1**

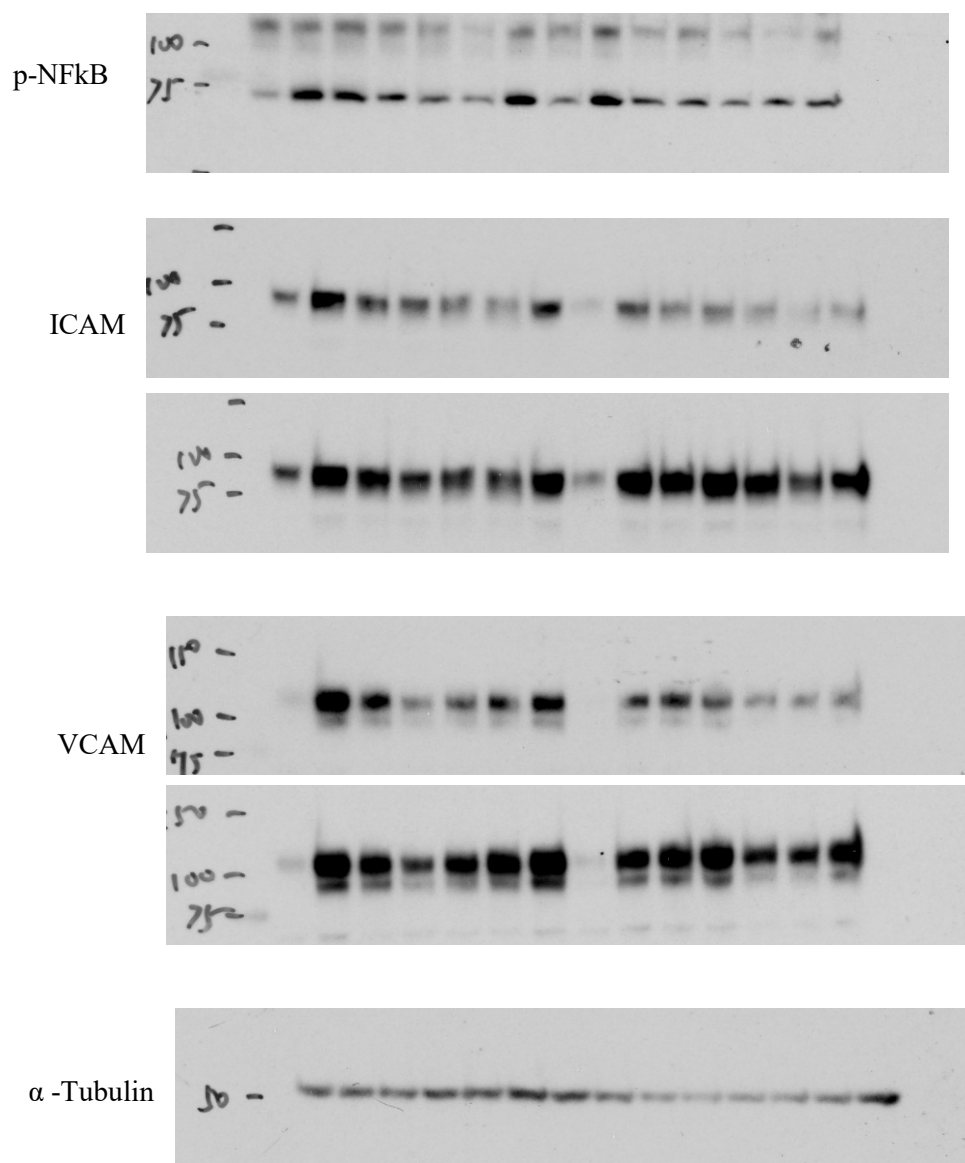

Supplement: Supplementary file 7 — Supplementary Information 7. [file 41598_2025_2582_MOESM7_ESM.pdf]

**Figure 4A-2**

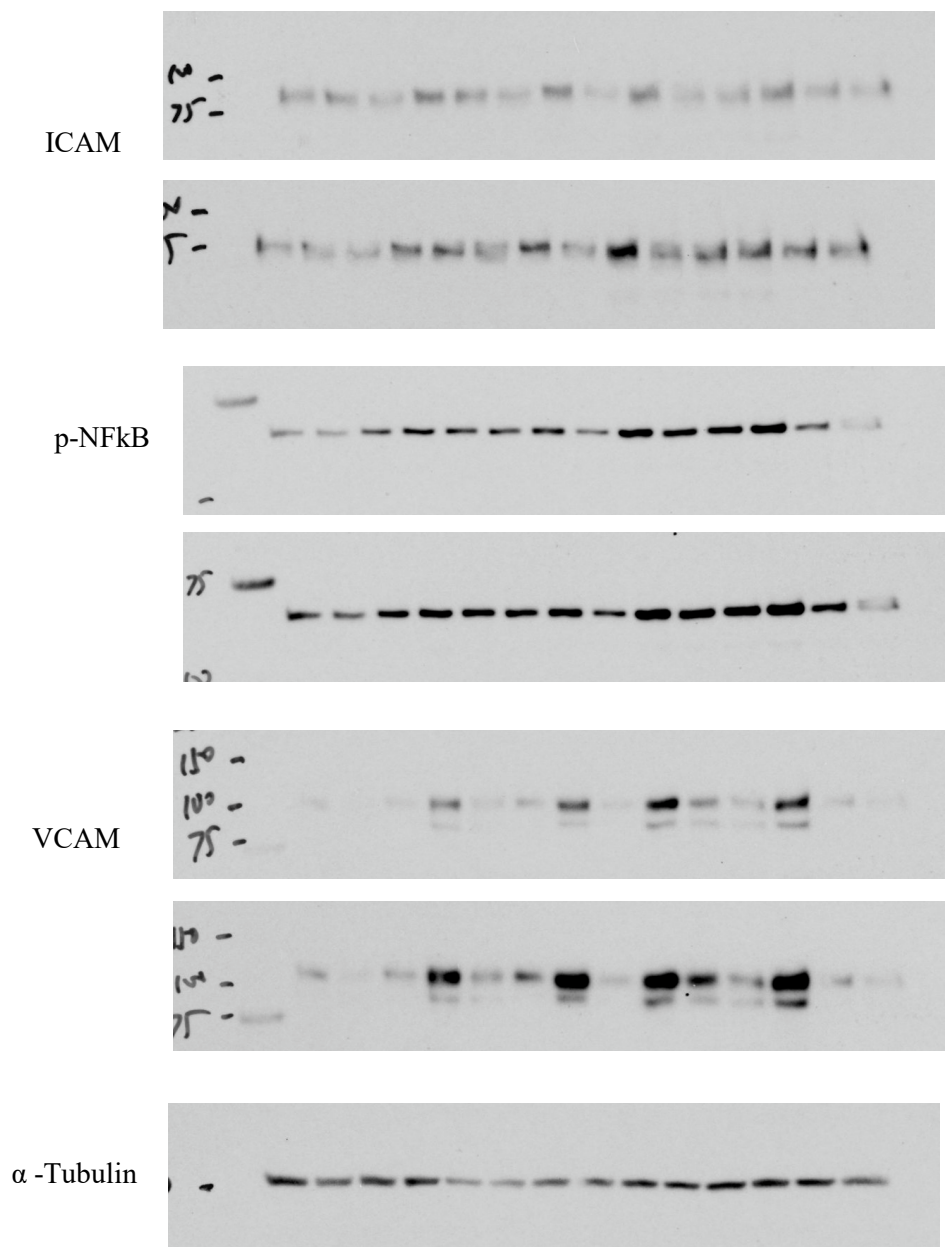

Supplement: Supplementary file 8 — Supplementary Information 8. [file 41598_2025_2582_MOESM8_ESM.pdf]

**Figure 5A**

ICAM-1

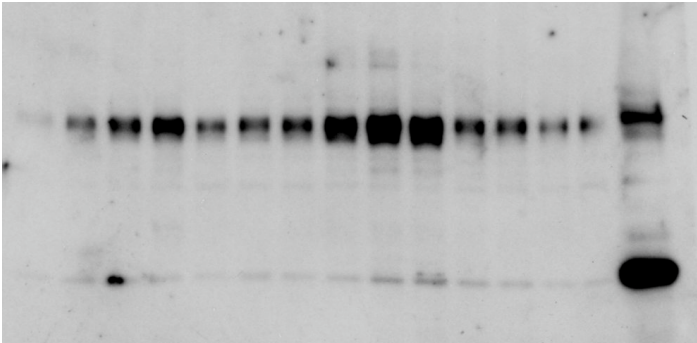

$\alpha$ -Tubulin

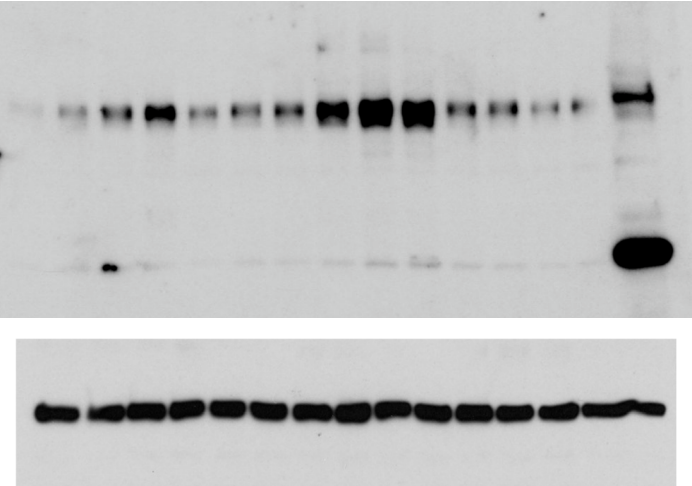

**Figure 5B**

p-NF $\kappa$ B

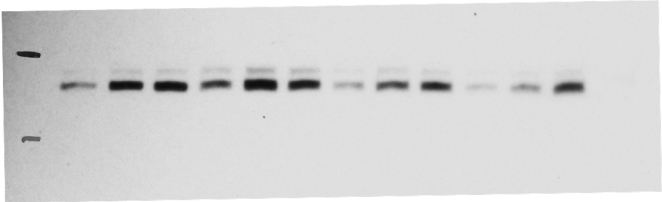

ICAM-1

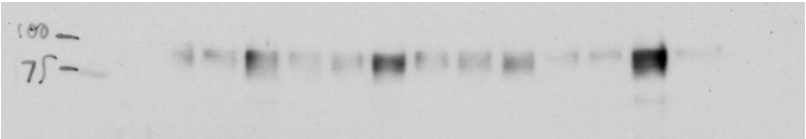

VCAM-1

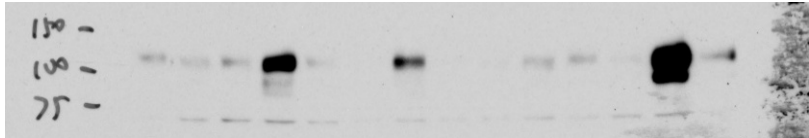

GFP-GPR68

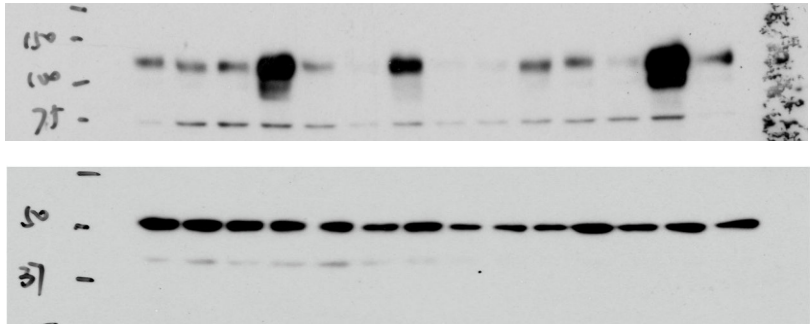

$\alpha$ -Tubulin

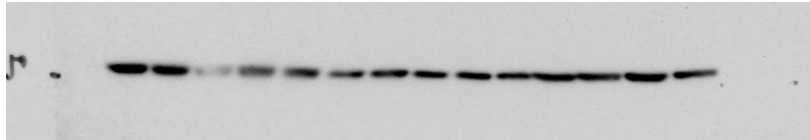

Supplement: Supplementary file 9 — Supplementary Information 9. [file 41598_2025_2582_MOESM9_ESM.pdf]

**Figure 6B**

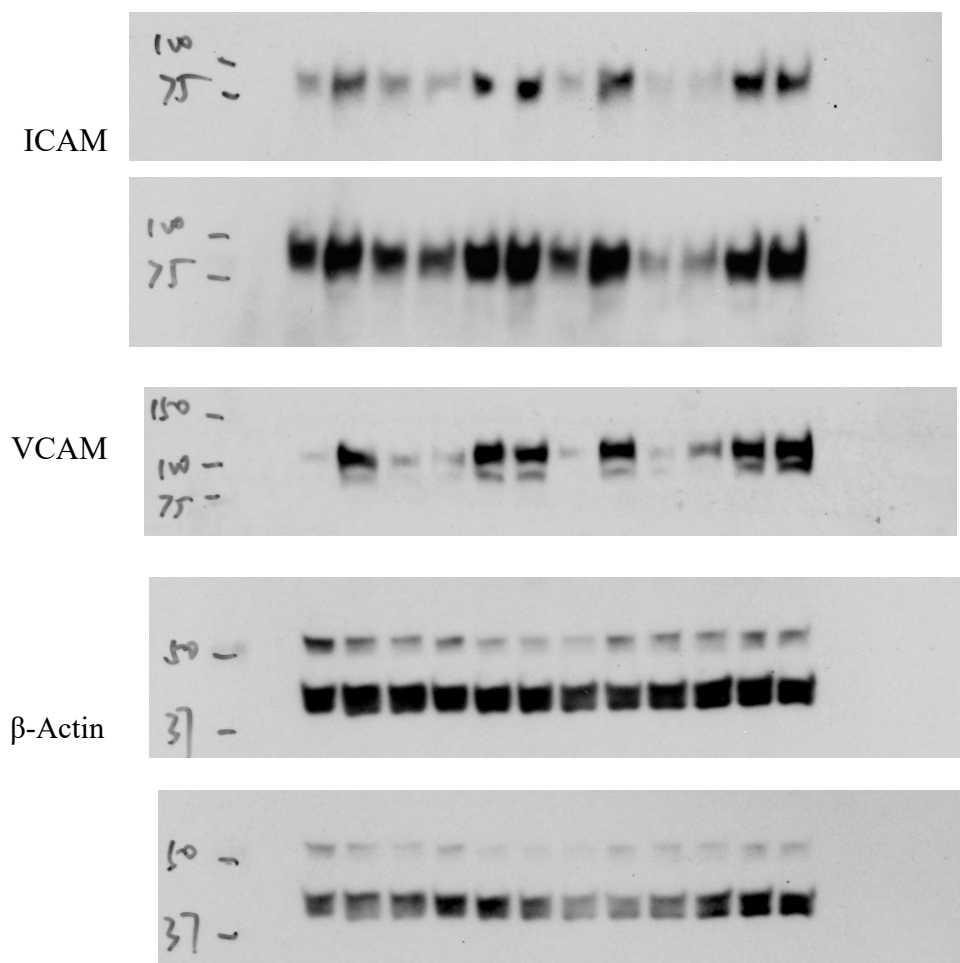

Supplement: Supplementary file 10 — Supplementary Information 10. [file 41598_2025_2582_MOESM10_ESM.pdf]

### Figure 7B

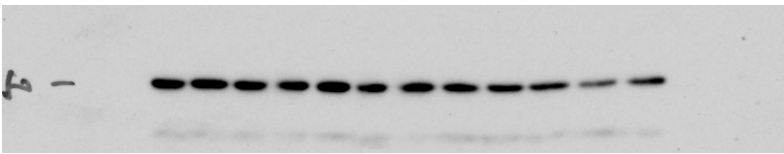

Supplement: Supplementary file 11 — Supplementary Information 11. [file 41598_2025_2582_MOESM11_ESM.pdf]

**Figure S3C**

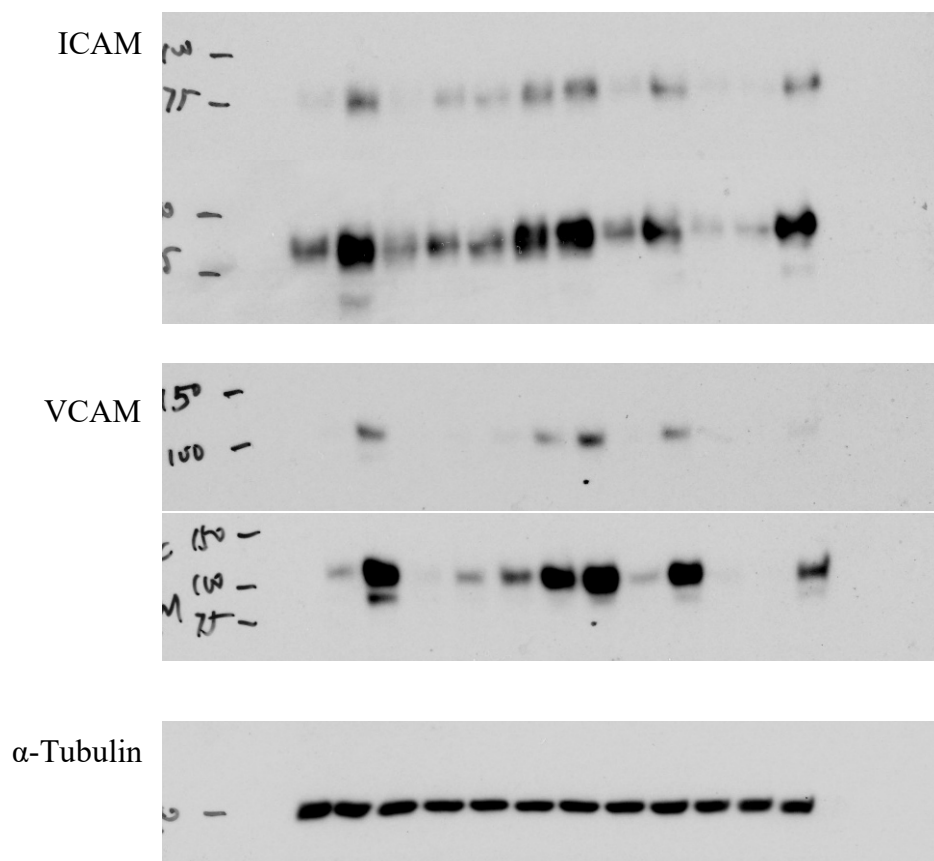

Supplement: Supplementary file 12 — Supplementary Information 12. [file 41598_2025_2582_MOESM12_ESM.pdf]

**Figure S4E**

**VCAM-1**

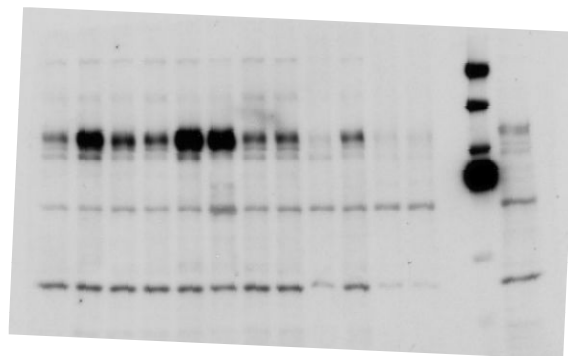

**$\alpha$ -Tubulin**

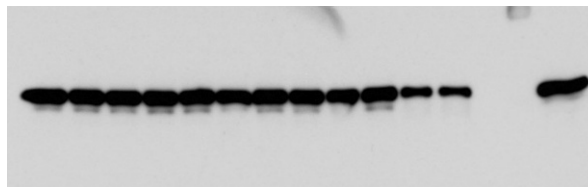

Supplement: Supplementary file 13 — Supplementary Information 13. [file 41598_2025_2582_MOESM13_ESM.pdf]
