# Supplementary material for: Novel small molecule inhibitor of GPR68 attenuates endothelial dysfunction and lung injury caused by bacterial lipopolysaccharide
Source: Sci Rep. 2025 Nov 5;15:38669. doi: 10.1038/s41598-025-02582-y (PMC12589648; doi:10.1038/s41598-025-02582-y)
Supplement: Supplementary file 5 — Supplementary Information 5. [file 41598_2025_2582_MOESM5_ESM.docx]

**Supplemental Figure Legends**

**Figure S1. (A)** Chemical structure of OGM-8345 is shown. **(B)** Cells challenged with LPS (50 ng/ml) with or without pretreatment with 3 µM OGM-8345 or 1 µM GPR4 inhibitor were loaded with Ca^2+^ indicator dye Flura-2 followed by exposure to acidic buffer to measure intracellular Ca^2+^. **(C)** Cells were treated with vehicle or OGM-8345 (3 µM, 3 h), and qPCR was carried out to determine mRNA expression levels of GPR68, GPR4, and GPR65.

**Figure S2. OGM attenuates LPS-induced inflammation in lung EC. (A, B)** HPAECs were exposed to indicated concentrations of OGM-8345 for 30 min prior to stimulation with 25 ng/ml LPS for 3h **(A)** or with OGM-8345 (5 µM) only for indicated time periods **(B)**. qPCR was performed to measure mRNA levels of endothelial pro-inflammatory marker genes. *p<0.05, vs. control and **p<0.05, vs. LPS, n=4.

**Figure S3. OGM-8345 inhibits CRX-induced endothelial dysfunction. (A)** HPAEC were pre-incubated with 3 µM of OGM-8345 for 30 min followed by addition of 50 ng/ml of CRX-527 for 6 h. Endothelial permeability for macromolecules was monitored by XPerT assay, counterstaining of cell nuclei was performed by DAPI. (**B**) HPAEC were exposed to CRX (50 ng/ml, 3 h) alone or pretreated with varying concentrations of OGM-8345 for 30 min. mRNA analysis of TNF-α, VCAM-1, ICAM-1, IL-6, IL-1β, and CXCL5 was done by qRT-PCR. *p<0.05 vs CRX only; n=4. (**C**) In post-treatment experiments, cells were stimulated with 50 ng/ml of CRX followed by addition of 3 µM of OGM at indicated time points; total treatment time is 6 h. Protein levels of ICAM-1 and VCAM-1 were determined by western blotting; probing for α-tubulin was used as a loading control.

**Figure S4. OGM-8345 attenuates LPS-induced endothelial dysfunction in lung microvascular endothelial cells. (A)** Human lung microvascular lung endothelial cells (HLMVEC, Lonza) were pre-incubated with OGM-8345 (3 µM, 30 min) followed by stimulation with LPS (100 ng/ml, 6 h) and immunostaining for VE-cadherin and F-actin. Arrows indicate paracellular gaps. Bar=10 µm. **(B)** Cells were treated with LPS (25 ng/ml) alone or in combination with Ogerin (10 µM) for 6 h followed by immunofluorescence analysis of VE-cadherin and F-actin. Arrows indicate paracellular gaps. Bar=10 µm. **(C)** EC barrier function was monitored by TER measurements over time in HLMVEC monolayers exposed to LPS (100 ng/ml) alone, pretreated with NE52 (1 µM), OGM-8345 (3 µM), or co-treated with Ogerin (10 µM). **(D)** Cells were pre-incubated with 3 µM of OGM-8345 or 1 µM of NE52 for 30 min followed by stimulation with LPS (50 ng/ml, 3 h). qPCR was performed to determine the mRNA expression of VCAM-1, ICAM-1, and IL-8. *p<0.05 vs LPS only, ND – no difference, n=3. **(E)** Cells were treated with indicated concentrations of inhibitors (30 min) followed by addition of 50 ng/ml of LPS for 6 h. VCAM-1 protein levels were determined by western blotting, α -tubulin was used as a loading control.
