# Supplementary material for: Novel small molecule inhibitor of GPR68 attenuates endothelial dysfunction and lung injury caused by bacterial lipopolysaccharide
Source: Sci Rep. 2025 Nov 5;15:38669. doi: 10.1038/s41598-025-02582-y (PMC12589648; doi:10.1038/s41598-025-02582-y)
Supplement: Supplementary file 14 — Supplementary Information 14. [file 41598_2025_2582_MOESM14_ESM.docx]

**Table S1. List of primer sequences used for RT-PCR**

| **Gene name** | **Forward Primer** | **Reverse Primer** |
| --- | --- | --- |
| Human TNF-α | AGGACGAACATCCAACCTTCCCAA | TTTGAGCCAGAAGAGGTTGAGGGT |
| Human VCAM-1 | CAGTAAGGCAGGCTGTAAAAGA | TGGAGCTGGTAGACCCTCG |
| Human ICAM-1 | TTGGGCATAGAGACCCCGTT | GCACATTGCTCAGTTCATACACC |
| Human IL-6 | CCTGAACCTTCCAAAGATGGC | TTCACCAGGCAAGTCTCCTCA |
| Human IL-8 | TGACTTCCAAGCTGGCCGTGG | ACTGCACCTTCACACAGAGCTGC |
| Human IL-1β | CTCGCCAGTGAAATGATGGCT | GTCGGAGATTCGTAGCTGGAT |
| Human CXCL5 | TGGACGGTGGAAACAAGG | CTTCCCTGGGTTCAGAGA |
| Human CXCL10 | GGAACCTCCAGTCTCAGCACC | GCGTACAGTTCTAGAGAGAGGTAC |
| Human E-selectin | GAA GGA TGG ACG CTC AAT GG | TGG ACT CAG TGG GAG CTT CAC |
| Human GAPDH | ATGGGGAAGGTGAAGGTC | GGGGTCATTGATGGCAACAATA |
| Mouse TNF-α | CTGTAGCCCACGTCGTAGC | TTGAGATCCATGCCGTTG |
| Mouse VCAM-1 | ACGAGGCTGGAATTAGCAGA | TCGGGCACATTTCCACAAG |
| Mouse ICAM-1 | CTGCCTCTGAAGCTCGGATA | GTCACCTCTACCAAGGCAGT |
| Mouse IL-6 | CCGGAGAGGAGACTTCACAG | TCCACGATTTCCCAGAGAAC |
| Mouse IL-1β | GAAATGCCACCTTTTGACAGTG | TGGATGCTCTCAGGACAG |
| Mouse KC | GCGCCCAAACCGAAGTCATA | ATGGGGGATGCAGGATTGAG |
| Mouse CXCL2 | CGCTGTCAATGCCTGAAGAC | ACACTCAAGCTCTGGATGTTCTTG |
| Mouse GAPDH | AATGTGTCCGTCGTGGATCT | AGACAACCTGGTCCTCAGTG |
